# Topic modeling for cluster analysis of large biological and medical datasets

**DOI:** 10.1186/1471-2105-15-S11-S11

**Published:** 2014-10-21

**Authors:** Weizhong Zhao, Wen Zou, James J Chen

**Affiliations:** 1 Division of Bioinformatics and Biostatistics, National Center for Toxicological Research, U.S. Food and Drug Administration, Jefferson, Arkansas 72079, USA; 2 College of Information Engineering, Xiangtan University, Xiangtan, Hunan Province, China

**Keywords:** Topic modeling, cluster analysis, large biological and biomedical datasets, data mining

## Abstract

**Background:**

The big data moniker is nowhere better deserved than to describe the ever-increasing prodigiousness and complexity of biological and medical datasets. New methods are needed to generate and test hypotheses, foster biological interpretation, and build validated predictors. Although multivariate techniques such as cluster analysis may allow researchers to identify groups, or clusters, of related variables, the accuracies and effectiveness of traditional clustering methods diminish for large and hyper dimensional datasets. Topic modeling is an active research field in machine learning and has been mainly used as an analytical tool to structure large textual corpora for data mining. Its ability to reduce high dimensionality to a small number of latent variables makes it suitable as a means for clustering or overcoming clustering difficulties in large biological and medical datasets.

**Results:**

In this study, three topic model-derived clustering methods, highest probable topic assignment, feature selection and feature extraction, are proposed and tested on the cluster analysis of three large datasets: *Salmonella *pulsed-field gel electrophoresis (PFGE) dataset, lung cancer dataset, and breast cancer dataset, which represent various types of large biological or medical datasets. All three various methods are shown to improve the efficacy/effectiveness of clustering results on the three datasets in comparison to traditional methods. A preferable cluster analysis method emerged for each of the three datasets on the basis of replicating known biological truths.

**Conclusion:**

Topic modeling could be advantageously applied to the large datasets of biological or medical research. The three proposed topic model-derived clustering methods, highest probable topic assignment, feature selection and feature extraction, yield clustering improvements for the three different data types. Clusters more efficaciously represent truthful groupings and subgroupings in the data than traditional methods, suggesting that topic model-based methods could provide an analytic advancement in the analysis of large biological or medical datasets.

## Background

Recent advances in biotechnology have generated massive amounts of biological and medical data for disease diagnosis/prognosis, unknown compound toxicity prediction, and pathogen identification in outbreak investigation, etc. Identification of pattern and structure among a large number of samples and/or the associated variables requires the development of more powerful statistical methods and data mining techniques. For example, genomic microarray and proteomic technologies are often used to identify genes and proteins that have similar functionality for understanding biological processes or identifying new biomarkers for targeted therapy, etc. [1-6]. Data mining techniques have been developed to classify patients into distinct subgroups for treatment assignment by identifying sets of genomic markers of individual patients. In food safety surveillance, the PulseNet managed by the Center of Disease Control (CDC) (http://www.cdc.gov/pulsenet) has been using the pulsed-field gel electrophoresis (PFGE) for the source tracking of foodborne pathogens [7-9]. PulseNet has collected more than 350,000 profiles of over 2,000 *Salmonella *serotypes. The fingerprint of an isolate is characterized by the presence or absence at designated band locations in PFGE analysis. Classification models were developed to characterize and identify serotype of isolates in outbreak investigation from the analysis of PFGE fingerprint [8]. The FDA Adverse Event Reporting System (FAERS) database is the primary database for post-marketing safety surveillance of all approved drugs and therapeutic biologic products. The FAERS database consists of over 5,000 drugs and over 16,000 adverse events reported. Data mining methods have been proposed to detect signals of unexpected occurrences in FAERS [10-12].

A dataset can be expressed in a two-way data matrix with rows representing samples and columns representing the measured variables that characterize the corresponding samples. A large dataset may have a large number of samples, such as the PFGE dataset of *Salmonella *or other foodborne pathogens [8,9]; or a large number of variables, such as a microarray dataset [13,14]. The analysis of large amounts of multivariate data to discover the hidden patterns and the relationships between patterns presents big challenges in both analysis methodology and data interpretation.

Cluster analysis is a commonly used data mining technique to explore the relationships among attributes, samples and the relationships between attributes and samples. Clustering algorithms assign samples or attributes to clusters based on their similarity. Cluster analysis can be used as a preliminary method for classification or for finding new classes. Hierarchical clustering tree (HCT) [15] and *k*-means [16] are the two most popular clustering methods. HCT sequentially merges the most similar cluster sub-nodes resulting in a tree-like dendrogram. *K-*means is the most commonly used non-hierarchical clustering algorithm. In *k*-means clustering procedures, samples are divided into *k *partitions or clusters based on a measure of similarity. Unlike the hierarchical clustering, the number of clusters in a *k*-means analysis must be specified *a priori*. Simulation studies have shown that *k*-means algorithms and other non-hierarchical clustering algorithms perform poorly when random initial seeds are used; their performance is improved when the results from hierarchical methods are used to form the initial partition [17]. Thus, hierarchical and non-hierarchical techniques should be applied as complementary rather than as competing clustering techniques.

Topic modeling algorithms are statistical methods that analyze the words of documents to discover the themes that pervade a large collection of documents [18]. The basic idea of topic modeling is that a document is a mixture of latent topics and each topic is expressed by a distribution of words. Latent Dirichlet Allocation (LDA) is the most popular topic modeling method in the field of text mining. LDA is an enhanced version of earlier models [19,20] and uses two Dirichlet-Multinomial distributions to model the relationships between documents and topics and the relationships between topics and words. The output of LDA provides two probability matrices: 1) the (posterior) probability distribution of each document over the topics, and 2) the probability distribution of words in a given topic. The LDA analysis commonly uses approximate methods, such as variation inference [21] or Markov chain Monte Carlo (MCMC) [22], to calculate the posterior probabilities. The calculated probability matrixes are used to make inference about the topics and documents for text mining. LDA has been shown to be an effective tool for text mining of large datasets [23,24], and computational software is freely available [25].

In this study, we proposed to apply LDA topic modeling for cluster analysis of large datasets. Three different datasets were selected to represent various types of large biological or medical datasets. These large datasets were transformed into the files of documents on which the LDA algorithm was run and two matrices were generated for each dataset. Three different cluster analysis methods were then applied on the topic model-derived data matrixes of the three datasets, and the most accurate method for each type of dataset was determined. The applications of the topic model on various large datasets provide new approaches to improve the accuracy and efficacy of the subgroup identification and data mining.

## Materials and methods

### Datasets

Three large data sets were utilized to evaluate the proposed approaches in this study. The first dataset was the *Salmonella *PFGE genotyping data from CDC [8,9]. It included 41,232 PFGE profiles of *Salmonella *outbreak-related isolates. The dataset contained 20 most common *Salmonella *serotypes and about 2,000 isolates for each of 20 serotypes. Each profile used 1/ 0 to represent the presence/absence of the electrophoresis bands, and each of 41,232 profiles was nominated to have 60 bands in the dataset. As a standard typing method used in *Salmonella *outbreak investigations, PFGE has been used by many laboratories to determine strain relatedness and confirm an outbreak of a bacterial disease by comparing the band profiles [8,9]. The serotype information of each profile was considered as the true label to evaluate the clustering results.

The second dataset was the public lung cancer microarray dataset originally collected from the Gene Expression Omnibus [14,26]. The dataset consisted of 111 lung cancer samples harboring 53 adenocarcinoma and 58 squamous cell carcinoma subtypes. Each sample was expressed by 54,613 continuous valued variables. The subtype of each sample was considered as the true label to evaluate the clustering results.

The third dataset was the breast cancer microarray dataset originally collected by van 't Veer et al. [13]; there were of 24,481 continuous valued gene expression variables from 97 patients. In this work, the data of the patient with "ID54" was removed from the dataset because it had 10,896 (about 44.5%) missing variables. The incomplete variables were also removed from the dataset. The final dataset consisted of 96 patients with 21,907 genomic variables. Although there were no true labels for the samples in breast cancer dataset, we used the survival analysis [27] to evaluate the clustering results for this dataset.

### Data preprocessing

In this step, each isolate/sample was transformed into one document and all documents constituted one corpus. For the *Salmonella *dataset, the PFGE bands were viewed as the words. Each isolate had its corresponding document consisting of the bands present, which had value 1 in the PFGE dataset. After the data preprocessing, the corpus of the *Salmonella *dataset contained 41,232 documents, where each document contained at most 60 words.

In both of lung and breast cancer microarray datasets, the expression value for each variable (gene) was normalized to 0 (smaller than the median value) or 1 (larger or equal than the median value) based on its median value. Each sample was transformed into one document. The variables with value 1 were considered as the words in the documents. The final corpus of the lung cancer dataset contained 111 documents and each document contained at most 54,613 words. The final corpus of the breast cancer dataset contained 96 documents and each document contained at most 21,907 words.

### Topic modeling

For a given dataset, topic modeling with LDA is utilized to model the relationships between samples and variables. LDA assumes that the dataset is generated by the following process [21]:

1. Pick a Multinomial distribution *φ_k _*(*k*∈{1,...,*K*}) for each topic from a Dirichlet distribution with hyper parameter *β*;

2. Pick a Multinomial distribution *θ_s _*(*s*∈{1,...,*S*}, where *S *is the number of samples in the dataset) for each sample from a Dirichlet distribution with parameter *α*;

a) Pick a topic *z *from a Multinomial distribution with hyper parameter *θ_s_*.

b) Pick a word *w_n _*(*n*∈{1,...,*N*}, where *N *is the number of words in the current document) from a Multinomial distribution with parameter *φ_z_*.

Based on the generative process above, the probability of a given dataset *D *= {*D*_1_,..., *D_S_*} is formalized as

$$p\left(D|\alpha ,\beta \right)=\overset{s=S}{\underset{s=1}{\prod }}\int p\left(D_{s}|\varphi ,\theta_{s}\right)p\left(\varphi |\beta \right)p\left(\theta_{s}|\alpha \right)d\theta_{s}$$

**Figure 1:**
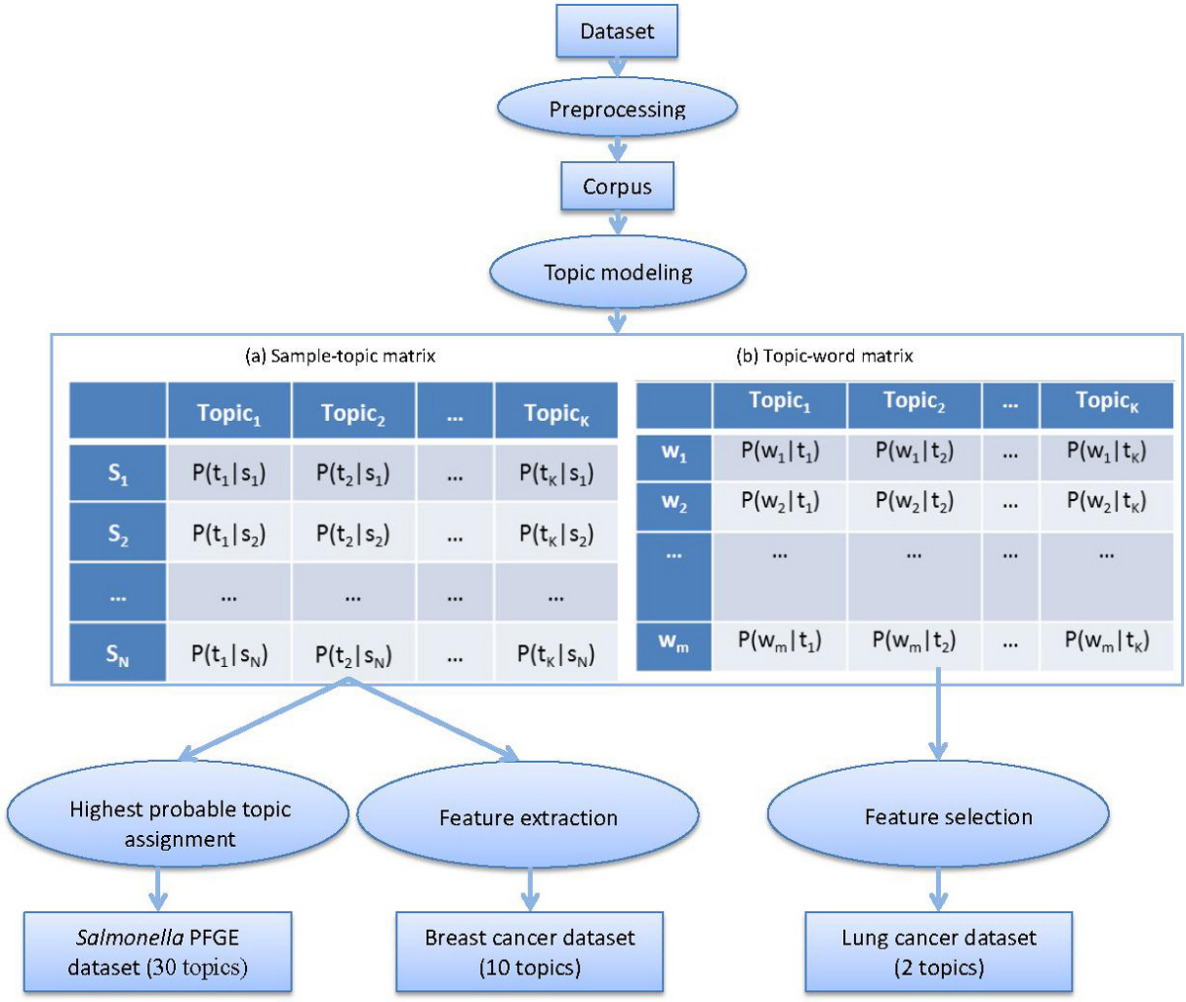
**The workflow of the topic model-derived clustering methods**.

Through maximizing the probability, LDA derives the posterior distributions of *θ *(the matrix in Figure 1a) and *φ *(the matrix in Figure 1b) which are used for cluster analysis in our study.

The LDA program implemented in Mallet [25] was applied for topic modeling. In Mallet, Gibbs sampling [24], a special case of MCMC approach, was utilized to calculate the two matrices in Figure 1. The number of iterations was set to 2000 in Gibbs sampling and other parameters were set to default values in Mallet in all calculations.

As shown in Figure 1, the sample-topic matrix (Figure 1a) depicts the distribution of the topics in the documents (samples). Each row has a mixture of topics and represents one document. Each entry gives the probability of the corresponding topic in the document, where the sum of probabilities in each row is 1. The topic-word matrix (Figure 1b) depicts the distribution of the words in a given topic. Each column of the matrix gives the probable distributions of the words in each topic, where the sum of probabilities in each column is 1.

### Cluster analysis methods

Three *topic model-derived *c*lustering *methods were proposed based on the two LDA-derived matrices.

#### 1. Topic model-derived clustering based on highest probable topic assignment

The method was based on sample-topic matrix (Figure 1a) and called "highest probable topic assignment". In this method, the LDA-derived topics were made as the clusters of the dataset. Then, each sample was assigned to the cluster (Topic) with the highest probability in the row of the sample-topic matrix.

#### 2. Topic model-derived clustering based on feature extraction

In this method, LDA was utilized as a feature extraction approach for cluster analysis. The LDA-derived topics were considered as the new features of datasets. The sample-topic matrix (Figure 1a) was treated as a new representation of the original dataset. Based on the sample-topic matrix, conventional clustering algorithms, such as *k*-means and hierarchical clustering algorithms were used for the clustering analysis.

#### 3. Topic model-derived clustering based on feature selection

In this method, the topic-word matrix (Figure 1b) was used for feature selection. The words with high probabilities in each LDA-derived topic were selected to express the dataset. Therefore, a reduced dataset with selected words (variables) was generated, based on which the conventional clustering could be conducted. In this study, the top 50 high probability words were chosen in each topic.

### Hierarchical cluster analysis

For each of the 30 clusters, the average of PFGE band presence (value of 1) /absence (value of 0) of all the sample isolates at 60 designated band locations was calculated as the characteristic mean of the corresponding cluster. Then, the complete-link hierarchical clustering algorithm was applied on the Euclidean distance measures to investigate the relationships among the 30 clusters.

### Survival analysis

For the breast cancer dataset, based on the obtained clusters (groups) and survival time information of the samples, the survival package in R was utilized for survival analysis. Specifically, function "survfit" was used to generate the Kaplan-Meier curves [28] for the patients in the clusters and function "survdiff" was used for the logrank test [29] for differences among clusters.

### Normalized mutual information (NMI)

Normalized mutual information [30] was utilized to evaluate the clustering results. NMI is an external validation metric to evaluate the quality of clustering result with respect to the given true labels of the datasets. If random variable *Z*' denotes the cluster assignments of instances in obtained clustering result, and random variable *Z *denotes the true class labels, then NMI is defined as follows:

$$NMI=\frac{I\left(Z^{\prime };Z\right)}{\left(H\left(Z^{\prime }\right)+H\left(Z\right)\right)/2}$$

Where *I*(*Z*';*Z*) = *H*(*Z*) - *H*(*Z*|*Z*') is the mutual information between the random variables *Z*' and *Z, H*(*Z*) is the Shannon entropy of *Z*, and *H*(*Z*|*Z*') is the conditional entropy of *Z *given *Z*' [31]. The range of NMI values is 0-1. In general, the larger the NMI value is, the better the clustering quality is.

## Results

In this study, three large datasets representing different types of large biological or medical were selected to illustrate the applications of topic modeling for cluster analysis. The LDA algorithm transformed the original datasets into the files of documents and generated two matrices for each of the three datasets. Three different topic model-derived clustering methods were applied to the LDA-derived matrices from the three large datasets. After the result comparison (data not shown here), the best-fitting cluster analysis method was selected on the basis of the most biological accuracies for each dataset.

### Analysis of *Salmonella *PFGE dataset

Topic model-derived clustering based on highest topic assignment.

The topic model-derived clustering based on highest topic assignment yielded the most accurate classification results for the *Salmonella *PFGE dataset, as compared to the other two topic model-derived clustering methods (Table S1 in Additional file 1). The LDA algorithm was run on the 41,232 PFGE profiles of 20 serotypes with 30 topics (Table 1). The 30 topics representing 30 clusters were labelled with the serotypes of dominant isolates in the clusters (first column in Table 1). The percentages of the most dominant serotype for each of 30 clusters were also calculated (fourth column in Table 1). In 24 out of 30 clusters, the percentages of the most dominant serotypes were greater than 75%. The exceptions fell in the clusters T8 labelled as serotype Muenchen with 36.60%, T6 and T20 as serotype Typhimurium var. 5-, and T0, T21 and T24 as Typhimurium.

**Table 1:** Cluster analysis of the *Salmonella *dataset using the method of topic model-derived clustering based on highest probable topic assignment.

Most dominant serotype	Number of isolates	Topic ID	% of most dominant serotype
Enteritidis	1046	T11	99.71%
Saintpaul	989	T12	99.60%
Paratyphi B	850	T26	99.41%
Enteritidis	1236	T2	99.35%
Saintpaul	709	T29	99.29%
Hadar	1837	T18	99.18%
Poona	1216	T22	98.68%
Oranienburg	1847	T27	98.65%
Poona	504	T16	98.41%
Newport	1179	T15	98.39%
Braenderup	852	T14	98.12%
Heidelberg	2125	T23	96.80%
Typhi	1845	T19	95.88%
Braenderup	1135	T9	95.51%
Javiana	2002	T1	94.36%
Agona	1846	T13	91.87%
Infantis	2130	T25	89.48%
Thompson	2195	T7	89.25%
4, 5, 12:i-	1024	T28	86.82%
Paratyphi B	1041	T10	85.40%
Typhimurium var. 5-	288	T5	84.03%
Montevideo	2240	T17	80.31%
4, 5, 12:i-	854	T3	79.39%
Mississippi	1860	T4	78.60%
Typhimurium var. 5-	1201	T20	66.36%
Typhimurium	1217	T21	54.97%
Typhimurium	738	T0	51.63%
Typhimurium var. 5-	417	T6	48.68%
Typhimurium	815	T24	38.16%
Muenchen	3994	T8	36.60%

**Figure 2:**
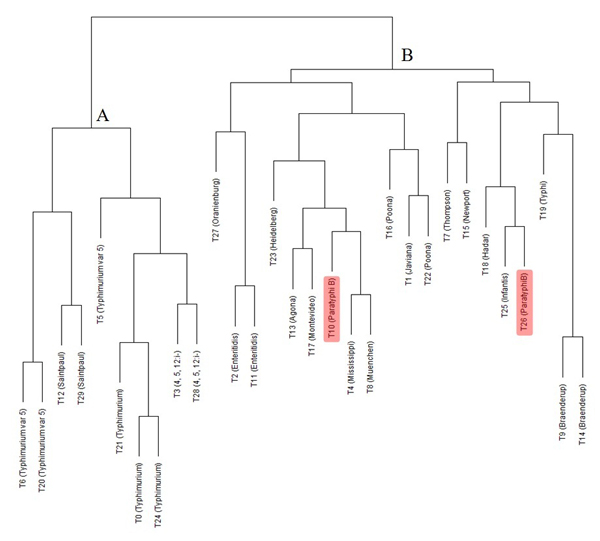
**Hierarchical cluster analysis of the LDA-derived clusters**. The dendrogram shows a simplified tree-structure of 30 topic clusters (T0 to T29). For each of the 30 clusters, the average of PFGE band presence /absence of all sample isolates at 60 designated band locations were calculated. The hierarchical clustering algorithm was applied on the Euclidean distance measures of the means of the 30 clusters and two major clusters (A and B) were grouped.

To further investigate the relationships between the 30 clusters, the complete-link hierarchical clustering analysis was conducted on the Euclidean distance measures of the characteristic means of 30 clusters (Material and Methods). In the resultant Figure 2 dendrogram tree, most of the clusters labelled with the same serotypes grouped together, such as the two clusters of Braenderup (T9 and T14), two clusters of Enteritidis (T2 and T11), three clusters of Typhimurium (T0, T21, and T24), two clusters of 4,5,12:i- (T3 and T28), two clusters of Saintpaul (T12 and T29), and two clusters of Typhimurium var. 5- (T6 and T20). The only exception was the two clusters (T10 and T26 highlighted in red) of Paratyphi B that classified into two different branches, indicating that the serotype Paratyphi B might have two subtypes.

### Analysis of the lung cancer dataset

Topic model-derived clustering based on feature selection.

The topic model-derived clustering based on selection of the highest probability features emerged as the best fitting method for the lung cancer dataset after comparison with the other two methods (Table 2). In this method, a pre-specified fixed number of words (features) with highest probability in each topic were selected. The results optimized when the topic number was set to 2 and the number of features as 50. Under this parameter setting, the 54,613 variables of each of the 111 samples in the lung cancer dataset were reduced to 100 selected features by the LDA algorithm. The selected genes were listed in Table S3 in Additional file 1. *K*-means was then applied to the 100 features for the clustering analysis. The results were compared with two conventional methods, *k*-means and PCA [32]. The conventional *k*-means algorithm was directly applied to the original 54,613 continuous-valued variables, while the PCA method [32] was first used to reduce the original 54,613 variables to 10 features followed by the *k*-means algorithm. Table 3 compares results for *k *set as 2, 3 and 4.

**Table 2:** Comparison of the results on the lung cancer dataset using the three proposed topic model-derived clustering methods.

Methods	*k*	Cluster ID	Adenocarcinoma	Squamous cell carcinoma	No. of misclassified samples	NMI
Clustering based on feature selection	2	1	42	11	**22**	**0.2809**
		2	11	47		
	
	3	1	40	8	**21**	**0.2417**
		2	4	15		
		3	9	35		
	
	4	1	37	8	**18**	**0.2926**
		2	9	35		
		3	0	14		
		4	7	1		

Clustering based on highest topic assignment	2	1	13	46	25	0.2296
		2	40	12		
	
	3	1	11	29	25	0.1847
		2	37	9		
		3	5	20		
	
	4	1	5	13	26	0.1744
		2	13	26		
		3	1	12		
		4	34	7		

Clustering based on feature extraction	2	1	13	47	24	0.2461
		2	40	11		
	
	3	1	8	34	24	0.2055
		2	8	16		
		3	37	8		
	
	4	1	7	6	25	0.1820
		2	33	6		
		3	8	31		
		4	5	15		

**Table 3:** Comparison of the results on the lung cancer dataset using the proposed method of topic model-derived clustering based on feature selection and two conventional clustering methods of *k*-means and PCA.

Methods	*k*	Cluster ID	Adenocarcinoma	Squamous cell carcinoma	No. of misclassified samples	NMI
Topic model-derived clustering based on feature selection	2	1	42	11	**22**	**0.2809**
		2	11	47		
	
	3	1	40	8	**21**	**0.2417**
		2	4	15		
		3	9	35		
	
	4	1	37	8	**18**	**0.2926**
		2	9	35		
		3	0	14		
		4	7	1		

***k***-means	2	1	41	12	24	0.2461
		2	12	46		
	
	3	1	8	35	31	0.1365
		2	27	17		
		3	18	6		
	
	4	1	6	14	25	0.1602
		2	22	6		
		3	18	6		
		4	7	32		

PCA (10 features) + ***k***-means	2	1	12	46	24	0.2461
		2	41	12		
	
	3	1	8	35	31	0.1456
		2	22	6		
		3	23	17		
	
	4	1	16	5	25	0.1605
		2	6	14		
		3	7	32		
		4	24	7		

The fourth and fifth columns in Table 3 give the numbers of the two sample subtypes Adenocarcinoma and Squamous cell carcinoma in each cluster, respectively. Each cluster was labelled as the subtype having the most prevalent samples in the cluster. Two criteria, the number of misclassified samples and normalized mutual information (NMI) [30], were utilized to evaluate the clustering results. NMI was used to compare the difference between the clustering result obtained and the truth clusters in the dataset. The larger the NMI, the better of the clustering results. The results in Table 3 show that the proposed topic model-derived clustering using the feature selection method yields the best clustering on both criteria, as compared to the other two conventional cluster analysis methods. Specifically, for *k *set to 4 in *k*-means, our proposed method gives the best cluster results with only 18 samples misclassified.

### Analysis of the breast cancer dataset

Topic model-derived clustering based on feature extraction.

The 96 patients from the breast cancer dataset were best clustered using the method based on feature extraction. The topic number was set as 10 for feature extraction; the *k*-means was then applied to the 10 derived features.

**Figure 3:**
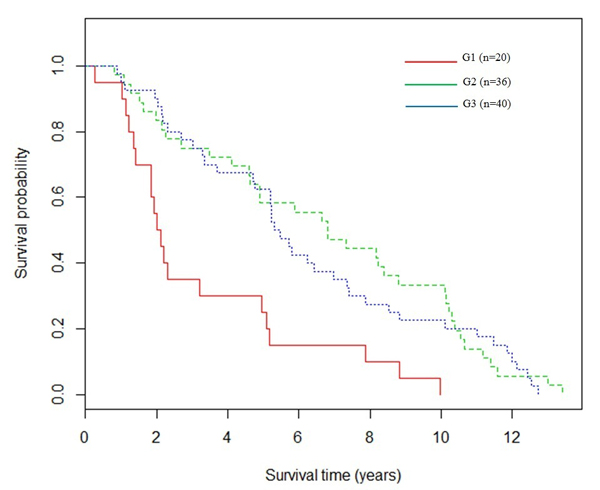
**Survival analysis of the breast cancer dataset *k *was set as 3**. The three subgroups were represented by three colors, respectively. The p-value of the logrank test for the differences among the three subgroups was 0.000174; the test for the differences between G2 and G3 was calculated as 0.645.

Survival analysis was conducted to evaluate the clustering results. For *k *= 3, the 96 breast cancer patients were clustered into three groups, G1 (*n *= 20), G2 (*n *= 36), and G3 (*n *= 40). The Kaplan-Meier survival curves of the three clusters were shown in Figure 3. The p-value of the logrank test for the differences among the three groups was 0.000174 (Material and Methods). However, the survival curves of G2 and G3 were similar, and the p-value of the logrank test between G2 and G 3 was 0.645, indicating non-significant difference in survival between these two patient groups.

**Figure 4:**
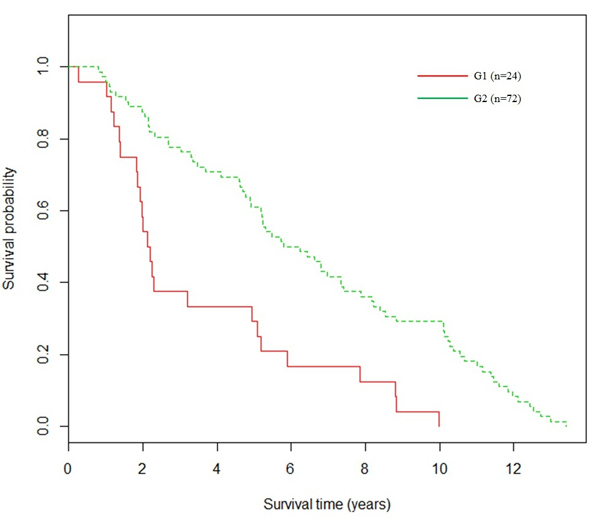
**Survival analysis of the breast cancer dataset *k *was set as 2**. The two subgroups were represented by two colors, respectively. The p-value of the logrank test for difference among the two subgroups was 4.6e-5.

For *k *= 2, the 96 patients were clustered into G1 (*n *= 24) and G2 (*n *= 72). The patients in G3 from the 3-means cluster analysis (Figure 3) were divided into two parts: four patients were grouped into G1 (Figure 4) and 36 patients were grouped into G2 (Figure 4). Figure 4 shows two distinguishable survival curves of G1 and G2, where the p-value for the logrank test is 4.6e-5, indicating significant differences between the two groups. From results of the 96 breast cancer patients, we can conclude that G1is the higher risk group.

## Discussion

Due to the advances in biotechnology and information technology, biological and medical datasets are growing rapidly in size and complexity and, consequently becoming increasingly difficult to process and analyze using traditional data mining methods. Multivariate techniques such as cluster analysis may allow researchers to identify groups, or clusters, of related variables. Reducing the dimension of a large dataset to a few clusters makes it possible to use standard statistical tools for all subsequent analyses.

Data mining has been used as a process that deals with the discovery of hidden knowledge and unexpected patterns, particularly the discovery of optimal clusters and interesting irregularities in large data bases. Topic modeling is an active research field in machine learning and has been widely used as an analytical tool to interpret large datasets in text mining [19-21,24] and image retrieval [23,33]. Here we applied topic modeling in a different way to reduce the dimensions of large datasets to yield more effective clustering analysis in various biological and biomedical data.

We have proposed three topic model-derived clustering methods and evaluated the efficacies/effectiveness on datasets from three different application fields. It was found that one method yielded better results than the others for each dataset (Table 3 Table S1 and S2 in Additional file 1). The topic model-derived clustering based on highest probable topic assignment used the LDA-derived topics as the clusters and the samples in the dataset were assigned to the clusters according to the highest probabilities. This method was found appropriate for the type of data with large number of samples but small number of variants, and with no causalities between the variants, such as *Salmonella *PFGE dataset. The results of this method on the PFGE dataset shown in Table 1 and Figure 2 not only reflected the biological understandings in concordance with the previous results [8,9], but also revealed some hidden patterns and interesting irregularities (see Results). Most of the serotypes were distinguishable and represented various topics. The low percentages of the serotype Muenchen in T8 reflected the biological fact that the PFGE patterns of serotype Muenchen were not unique and were very similar to those of other serotypes in topic T8. The five clusters (T0, T6, T20, T21, and T24) labelled as Typhimurium or Typhimurium var. 5- had less than 70% of the most dominant serotype, consistent with the fact that the serotypes 4,5,12:i- and Typhimurium var. 5- are variants of Typhimurium and isolates of the three serotypes shared similar PFGE patterns [9,34]. Two clusters of Paratyphi B (T10 and T26) separated into two distant sub-branches in the dendrogram tree of Figure 2, indicating the existence of hidden subtypes of the serotype.

The lung cancer and breast cancer datasets represent typical high dimensional microarray data with thousands of genes involved for each sample. For this type of high dimensional data with large samples and large variables, the proposed methods of topic model-derived clustering based on feature selection and on feature extraction, yielded more accurate results than the method based on highest probable topic assignment (Tables 2, 3 and S2, Figures 3 and 4). In these two methods, LDA algorithms effectively reduced the high dimensions in the original datasets to a small number of features from which the following traditional clustering algorithms were able to generate more accurate results. Both methods are appropriate for use on the high dimensional datasets, such as the microarray datasets. The differences between the two methods are generated from the fact that the method based on feature extraction works on the sample-topic matrix, while the method based on feature selection generates the results on topic-word matrix. Therefore, the selection of the most appropriate method also depends on the research applications.

The goal of personalized medicine requires stratifying subgroups of disease to tailor treatment to match individual characteristics, needs, and preferences of a patient subgroup during all stages of care, including prevention, diagnosis, treatment, and follow-up. There were two subtypes in the lung cancer dataset, adenocarcinoma and squamous cell carcinoma. Patients with different lung cancer subtypes need different therapies in clinical treatment. The proposed method of the topic model-derived clustering based on feature selection yield more effective clustering results on this dataset than the other two topic model-derived methods (Table 2), as well as the two conventional clustering methods, *k*-means and PCA (Table 3). The two topics obtained by LDA were considered as the representatives of the two subtypes of lung cancer. The method of topic model-derived clustering based on highest probable topic assignment, in which only one topic was used to describe the differences between samples, may not be appropriate to microarray datasets having tens of thousands of genes included in the samples. In the proposed method of topic model-derived clustering based on feature selection, 50 genes with the highest probability were selected to represent each topic, and all of the genes in two topics greatly reduced the dimensionality from 54,613 variables to 100 selected genes. The cluster analysis performed much better on the dimension-reduced dataset than the other methods in segregating lung cancer patients into the two subtypes (Table 3). The selected genes for each topic (Table S3 in Additional file 1) will be further analysed for subtype prediction and pathway identification.

Since there is no available subgroup information for breast cancer, we were trying to understand if there are hidden relationships in the dataset. The proposed method based on feature extraction worked on the sample-topic matrix and gives the best clustering results among the three proposed methods (Table S2 in Additional file 1). In personalized medicine, the prognostic predictors (biomarkers) are identified to predict overall course of disease outcome for treatment recommendation. The clinical endpoint of breast cancer dataset is the patient survival time. For this endpoint, prognostic biomarker signatures typically classify patients into high risk group and low risk group. The high risk group would be recommend to receive more aggressive treatment, and low risk groups to receive standard treatment or no treatment. The obtained results from this study yield potential prognostic predictors for treatment selection (Figures 3 and 4).

## Conclusions

Topic modeling could be beneficially applied to various large datasets from biological or medical research areas. Each of the three proposed topic model-derived clustering methods, highest probable topic assignment, feature selection, and feature extraction, yielded the best clustering results for a distinct type of dataset. The application of the topic modeling approach to cluster analysis of large datasets can greatly improve the accuracy and efficacy of subgroup identification, and the proposed three methods provide new approaches for data mining of large datasets in biological and biomedical research.

## Competing interests

The authors declare that they have no competing interests.

## Authors' contributions

WZ (Zhao) performed all the calculations and data analysis, and wrote the first draft of the manuscript. WZ and JC developed the methods, had the original idea, and guided the data analysis and presentation of results. WZ and JC collected and generated the data. All authors contributed to data verification, approach evaluation, and assisted with writing the manuscript. All authors read and approved the final manuscript.

## Disclaimer

The findings and conclusions in this article have not been formally disseminated by the US Food and Drug Administration (FDA) and should not be construed to represent the FDA determination or policy.

## Supplementary Material

Additional file 1 Click here for file
