# Supplementary material for: Topic modeling for cluster analysis of large biological and medical datasets
Source: BMC Bioinformatics. 2014 Oct 21;15(Suppl 11):S11. doi: 10.1186/1471-2105-15-S11-S11 (PMC4251039; doi:10.1186/1471-2105-15-S11-S11)
Supplement: Additional file 1 [file 1471-2105-15-S11-S11-S1.doc]

# Supplemental information.

# Table S1. Comparison of the results by NMI (normalized mutual information) on the *Salmonella* PFGE dataset using the three proposed topic model-derived clustering methods.

| *k* | highest topic assignment  (*K*= *k*) | feature extraction  (*K*=10) | feature selection  (*K*=5) |
| --- | --- | --- | --- |
| 20 | 0.7199 | 0.6948 | 0.5640 |
| 30 | **0.7602** | 0.7481 | 0.5758 |
| 40 | 0.7535 | 0.7530 | 0.5805 |
| 50 | 0.7503 | 0.7491 | 0.5692 |

Notes: *K*: number of topics. The best-fitting number of topics was selected for each method on the basis of the most biological accuracies.

# Table S2. Comparison of the results on the breast cancer dataset using the three proposed topic model-derived clustering methods and two conventional clustering methods of *k*-means and PCA+*k*-means based on the log rank test.

| *k* | *k*-means | PCA  +*k*-means | highest topic assignment  (*K*= *k*) | feature extraction  (*K*=10) | feature selection  (*K*=4) |
| --- | --- | --- | --- | --- | --- |
| 2 | 0.0014 | 0.0003 | 0.7600 | **4.60e-05** | 0.0040 |
| 3 | 0.2930 | 0.072 | 0.0700 | 0.0002 | 0.1500 |
| 4 | 0.0066 | 0.076 | 0.3000 | 0.0002 | 0.0760 |

Notes: *K*: number of topics. The best-fitting number of topics was selected for each method on the basis of the most biological accuracies. When using the method of PCA+*k*-means, PCA was first applied to the dataset to reduce the features to 20 for the best results, followed by traditional *k*-means for the clustering analysis.

Table S3. The metadata of 100 selected genes in two topics (T0 and T1) for lung cancer dataset (GEO accession No. of lung cancer dataset: GSE3141; <http://www.ncbi.nlm.nih.gov/geo/query/acc.cgi?acc=GSE3141>).

| **T0** | | | | **T1** | | | |
| --- | --- | --- | --- | --- | --- | --- | --- |
| **ID_Ref** | **Gene Name** | **Encoding Protein** | **NCBI Accession No.** | **ID_Ref** | **Gene Name** | **Encoding Protein** | **NCBI Accession No.** |
| 231315_at | NKX2-1 | NK2 homeobox 1 | NM_001079668 /// NM_003317 | 223480_s_at | MRPL47 | mitochondrial ribosomal protein L47 | NM_020409 /// NM_177988 |
| 228471_at | ANKRD44 | ankyrin repeat domain 44 | NM_001195144 /// NM_153697 | 224345_x_at | FAM162A | family with sequence similarity 162, member A | NM_014367 |
| 212951_at | GPR116 | G protein-coupled receptor 116 | NM_001098518 /// NM_015234 | 218009_s_at | PRC1 | protein regulator of cytokinesis 1 | NM_003981 /// NM_199413 /// NM_199414 |
| 205776_at | FMO5 | flavin containing monooxygenase 5 | NM_001144829 /// NM_001144830 /// NM_001461 | 217744_s_at | PERP | PERP, TP53 apoptosis effector | NM_022121 |
| 1566959_at |  |  |  | 211662_s_at | VDAC2 | voltage-dependent anion channel 2 | NM_001184783 /// NM_001184823 /// NM_003375 /// NR_033675 |
| 1557626_at |  |  |  | 210766_s_at | CSE1L | CSE1 chromosome segregation 1-like (yeast) | NM_001316 |
| 229866_at | STK32A | serine/threonine kinase 32A | NM_001112724 /// NM_145001 | 205644_s_at | SNRPG | small nuclear ribonucleoprotein polypeptide G | NM_003096 |
| 244654_at | MYO1G | myosin IG | NM_033054 | 203663_s_at | COX5A | cytochrome c oxidase subunit Va | NM_004255 |
| 235706_at | CPM | carboxypeptidase M | NM_001005502 /// NM_001874 /// NM_198320 | 202666_s_at | ACTL6A | actin-like 6A | NM_004301 /// NM_177989 /// NM_178042 |
| 235556_at | C5orf41 | chromosome 5 open reading frame 41 | NM_001168393 /// NM_001168394 /// NM_153607 | 201112_s_at | CSE1L | CSE1 chromosome segregation 1-like (yeast) | NM_001316 |
| 229867_at | BTBD9 | BTB (POZ) domain containing 9 | NM_001099272 /// NM_001172418 /// NM_052893 /// NM_152733 | 213726_x_at | TUBB2C | tubulin, beta 2C | NM_006088 |
| 228677_s_at | RASAL3 | RAS protein activator like 3 | NM_022904 | 221434_s_at | C14orf156 | chromosome 14 open reading frame 156 | NM_031210 |
| 223751_x_at | TLR10 | toll-like receptor 10 | NM_001017388 /// NM_001195106 /// NM_001195107 /// NM_001195108 /// NM_030956 | 222623_s_at | ZNF639 | zinc finger protein 639 | NM_016331 |
| 218322_s_at | ACSL5 | acyl-CoA synthetase long-chain family member 5 | NM_016234 /// NM_203379 /// NM_203380 | 212296_at | PSMD14 | proteasome (prosome, macropain) 26S subunit, non-ATPase, 14 | NM_005805 |
| 212646_at | RFTN1 | raftlin, lipid raft linker 1 | NM_015150 | 208821_at | SNRPB | small nuclear ribonucleoprotein polypeptides B and B1 | NM_003091 /// NM_198216 |
| 209619_at | CD74 | CD74 molecule, major histocompatibility complex, class II invariant chain | NM_001025158 /// NM_001025159 /// NM_004355 | 202330_s_at | UNG | uracil-DNA glycosylase | NM_003362 /// NM_080911 |
| 209515_s_at | RAB27A | RAB27A, member RAS oncogene family | NM_004580 /// NM_183234 /// NM_183235 /// NM_183236 | 201637_s_at | FXR1 | fragile X mental retardation, autosomal homolog 1 | NM_001013438 /// NM_001013439 /// NM_005087 |
| 209500_x_at | TNFSF12-TNFSF13 /// TNFSF13 | TNFSF12-TNFSF13 readthrough /// tumor necrosis factor (ligand) superfamily, member 13 | NM_001198622 /// NM_001198623 /// NM_001198624 /// NM_003808 /// NM_172087 /// NM_172088 /// NM_172089 | 201479_at | DKC1 | dyskeratosis congenita 1, dyskerin | NM_001142463 /// NM_001363 |
| 204687_at | PARM1 | prostate androgen-regulated mucin-like protein 1 | NM_015393 | 200903_s_at | AHCY | adenosylhomocysteinase | NM_000687 /// NM_001161766 |
| 203940_s_at | VASH1 | vasohibin 1 | NM_014909 | 200703_at | DYNLL1 | dynein, light chain, LC8-type 1 | NM_001037494 /// NM_001037495 /// NM_003746 |
| 1556277_a_at |  |  |  | 222392_x_at | PERP | PERP, TP53 apoptosis effector | NM_022121 |
| 1556055_at |  |  |  | 220942_x_at | FAM162A | family with sequence similarity 162, member A | NM_014367 |
| 57540_at | RBKS | ribokinase | NM_022128 | 214737_x_at | HNRNPC | heterogeneous nuclear ribonucleoprotein C (C1/C2) | NM_001077442 /// NM_001077443 /// NM_004500 /// NM_031314 |
| 228579_at |  |  |  | 226600_at | TMTC3 | transmembrane and tetratricopeptide repeat containing 3 | NM_181783 |
| 222756_s_at | ARRB1 | arrestin, beta 1 | NM_004041 /// NM_020251 | 225447_at | GPD2 | glycerol-3-phosphate dehydrogenase 2 (mitochondrial) | NM_000408 /// NM_001083112 |
| 208209_s_at | C4BPB | complement component 4 binding protein, beta | NM_000716 /// NM_001017364 /// NM_001017365 /// NM_001017366 /// NM_001017367 | 225268_at | KPNA4 | karyopherin alpha 4 (importin alpha 3) | NM_002268 |
| 242372_s_at | MFSD4 | major facilitator superfamily domain containing 4 | NM_181644 | 224437_s_at | VTA1 | Vps20-associated 1 homolog (S. cerevisiae) | NM_016485 |
| 242268_at | CELF2 | CUGBP, Elav-like family member 2 | NM_001025076 /// NM_001025077 /// NM_001083591 /// NM_006561 | 218286_s_at | RNF7 | ring finger protein 7 | NM_014245 /// NM_183237 |
| 240890_at | LOC643733 | caspase 4, apoptosis-related cysteine peptidase pseudogene | NR_034078 /// NR_034079 | 218271_s_at | PARL | presenilin associated, rhomboid-like | NM_001037639 /// NM_018622 |
| 240600_at |  |  |  | 214214_s_at | C1QBP | complement component 1, q subcomponent binding protein | NM_001212 |
| 238668_at |  |  |  | 212653_s_at | EHBP1 | EH domain binding protein 1 | NM_001142614 /// NM_001142615 /// NM_001142616 /// NM_015252 |
| 236921_at |  |  |  | 210052_s_at | TPX2 | TPX2, microtubule-associated, homolog (Xenopus laevis) | NM_012112 |
| 234974_at | GALM | galactose mutarotase (aldose 1-epimerase) | NM_138801 | 209608_s_at | ACAT2 | acetyl-CoA acetyltransferase 2 | NM_005891 |
| 232615_at |  |  |  | 209080_x_at | GLRX3 | glutaredoxin 3 | NM_006541 |
| 231747_at | CYSLTR1 | cysteinyl leukotriene receptor 1 | NM_006639 | 208836_at | ATP1B3 | ATPase, Na+/K+ transporting, beta 3 polypeptide | NM_001679 |
| 229779_at | COL4A4 | collagen, type IV, alpha 4 | NM_000092 | 207508_at | ATP5G3 | ATP synthase, H+ transporting, mitochondrial Fo complex, subunit C3 (subunit 9) | NM_001002258 /// NM_001190329 /// NM_001689 |
| 227354_at | PAG1 | phosphoprotein associated with glycosphingolipid microdomains 1 | NM_018440 | 207098_s_at | MFN1 | mitofusin 1 | NM_033540 |
| 227002_at | FAM78A | family with sequence similarity 78, member A | NM_033387 | 204957_at | ORC5 | origin recognition complex, subunit 5 | NM_001197292 /// NM_002553 /// NM_181747 |
| 226344_at | ZMAT1 | zinc finger, matrin-type 1 | NM_001011657 /// NR_036431 | 203816_at | DGUOK | deoxyguanosine kinase | NM_080916 /// NM_080918 |
| 222592_s_at | ACSL5 | acyl-CoA synthetase long-chain family member 5 | NM_016234 /// NM_203379 /// NM_203380 | 202095_s_at | BIRC5 | baculoviral IAP repeat-containing 5 | NM_001012270 /// NM_001012271 /// NM_001168 |
| 222357_at | ZBTB20 | zinc finger and BTB domain containing 20 | NM_001164342 /// NM_001164343 /// NM_001164344 /// NM_001164345 /// NM_001164346 /// NM_001164347 /// NM_015642 | 201636_at | FXR1 | fragile X mental retardation, autosomal homolog 1 | NM_001013438 /// NM_001013439 /// NM_005087 |
| 214191_at | ICA1 | islet cell autoantigen 1, 69kDa | NM_001136020 /// NM_004968 /// NM_022307 | 201486_at | RCN2 | reticulocalbin 2, EF-hand calcium binding domain | NM_002902 |
| 214032_at | ZAP70 | zeta-chain (TCR) associated protein kinase 70kDa | NM_001079 /// NM_207519 | 201115_at | POLD2 | polymerase (DNA directed), delta 2, regulatory subunit 50kDa | NM_001127218 /// NM_006230 |
| 213475_s_at | ITGAL | integrin, alpha L (antigen CD11A (p180), lymphocyte function-associated antigen 1; alpha polypeptide) | NM_001114380 /// NM_002209 | 200870_at | STRAP | serine/threonine kinase receptor associated protein | NM_007178 |
| 212912_at | RPS6KA2 | ribosomal protein S6 kinase, 90kDa, polypeptide 2 | NM_001006932 /// NM_021135 | 229742_at | C15orf61 | chromosome 15 open reading frame 61 | NM_001143936 |
| 212346_s_at | MXD4 | MAX dimerization protein 4 | NM_006454 | 209125_at | KRT6A | keratin 6A | NM_005554 |
| 206995_x_at | SCARF1 | scavenger receptor class F, member 1 | NM_003693 /// NM_145350 /// NM_145352 /// NR_028075 /// NR_028076 | 204855_at | SERPINB5 | serpin peptidase inhibitor, clade B (ovalbumin), member 5 | NM_002639 |
| 206118_at | STAT4 | signal transducer and activator of transcription 4 | NM_003151 | 201820_at | KRT5 | keratin 5 | NM_000424 |
| 205518_s_at | CMAH | cytidine monophosphate-N-acetylneuraminic acid hydroxylase (CMP-N-acetylneuraminate monooxygenase) pseudogene | NR_002174 /// NR_027626 | 202853_s_at | RYK | RYK receptor-like tyrosine kinase | NM_001005861 /// NM_002958 |
| 204424_s_at | LMO3 | LIM domain only 3 (rhombotin-like 2) | NM_001001395 /// NM_018640 | 218226_s_at | NDUFB4 | NADH dehydrogenase (ubiquinone) 1 beta subcomplex, 4, 15kDa | NM_001168331 /// NM_004547 |
